# Effects of Brazilian green propolis on proteinuria and renal function in patients with chronic kidney disease: a randomized, double-blind, placebo-controlled trial

**DOI:** 10.1186/s12882-019-1337-7

**Published:** 2019-04-25

**Authors:** Marcelo Augusto Duarte Silveira, Flávio Teles, Andressa A. Berretta, Talita R. Sanches, Camila Eleutério Rodrigues, Antonio Carlos Seguro, Lúcia Andrade

**Affiliations:** 1 Division of Nephrology, University of São Paulo School of Medicine, São Paulo, SP Av. Dr. Arnaldo, 455, 3° andar, sala 3310, CEP 01246-903 Brazil; 2 School of Medicine, Federal University of Alagoas, Maceió, Brazil; 3 Laboratory of Research, Development & Innovation, Apis Flora Industrial e Comercial Ltda, Ribeirão Preto, Brazil

## Abstract

**Background:**

Chronic kidney disease (CKD) is a public health problem worldwide, and proteinuria is a well-established marker of disease progression in CKD patients. Propolis, a natural resin produced by bees from plant materials, has anti-inflammatory, immunomodulatory, and anti-oxidant properties, as well as having been shown to have an antiproteinuric effect in experimental CKD. The aim of this study was to evaluate the impact of Brazilian green propolis extract on proteinuria reduction and the changes in the estimated glomerular filtration rate (eGFR).

**Methods:**

This was a randomized, double-blind, placebo-controlled study including patients with CKD caused by diabetes or of another etiology, 18–90 years of age, with an eGFR of 25–70 ml/min per 1.73 m^2^ and proteinuria (urinary protein excretion > 300 mg/day) or micro- or macro-albuminuria (urinary albumin-to-creatinine ratio > 30 mg/g or > 300 mg/g, respectively). We screened 148 patients and selected 32, randomly assigning them to receive 12 months of Brazilian green propolis extract at a dose of 500 mg/day (*n* = 18) or 12 months of a placebo (*n* = 14).

**Results:**

At the end of treatment, proteinuria was significantly lower in the propolis group than in the placebo group—695 mg/24 h (95% CI, 483 to 999) vs. 1403 mg/24 h (95% CI, 1031 to 1909); *P* = 0.004—independent of variations in eGFR and blood pressure, which did not differ between the groups during follow-up. Urinary monocyte chemoattractant protein-1 was also significantly lower in the propolis group than in the placebo group—58 pg/mg creatinine (95% CI, 36 to 95) vs. 98 pg/mg creatinine (95% CI, 62 to 155); *P* = 0.038.

**Conclusions:**

Brazilian green propolis extract was found to be safe and well tolerated, as well as to reduce proteinuria significantly in patients with diabetic and non-diabetic CKD.

**Trial Registration.**

(ClinicalTrials.gov number NCT02766036. Registered: May 9, 2016).

**Electronic supplementary material:**

The online version of this article (10.1186/s12882-019-1337-7) contains supplementary material, which is available to authorized users.

## Background

Chronic kidney disease (CKD) is a public health problem worldwide, and its prevalence has been increasing exponentially [1, 2]. Progression to more advanced stages of the disease is associated with high rates of morbidity and mortality, mainly due to cardiovascular diseases, and renal replacement therapies (dialysis and renal transplantation) present high costs to the health system [3–5].

In recent years, several clinical trials have been conducted to test the effect that certain drugs have on the progression of CKD [6–8]. However, since the first demonstrations of the antiproteinuric effect of angiotensin-converting enzyme (ACE) inhibitors and angiotensin receptor blockers (ARBs), there have been no studies evaluating any new class of drugs with the same impact on proteinuria or renal function [9–11].

Higher levels of proteinuria and albuminuria are associated with a more rapid decline in the glomerular filtration rate (GFR), as well as with a higher incidence of fatal and nonfatal cardiovascular events [9, 12–14]. Therefore, medications that have an antiproteinuric effect can minimize the risks of progression of CKD and consequently cardiovascular mortality [2, 15].

The health care system rationale, in terms of sustainability and greater accessibility, should involve a continuous search for greater knowledge and the development of new tools that are efficient and safe, as well as reducing costs. Natural products have recently come to play an important role in the development and discovery of new drugs [16, 17].

Propolis is a product derived from resins and plant exudates; its composition varies depending on the geographic region, flora, and local climate; and it is used by bees to protect the hive against macro- and micro-invaders [16, 18, 19]. Because of its specific chemical and biological characteristics, propolis has been used for hundreds of years by various peoples around the world, with diverse cultures, for medicinal purposes [16]. In recent decades, it has been shown to have antimicrobial, anti-inflammatory, immunomodulatory, antioxidant, and anticancer properties [19–22].

In one recent study [23], propolis was found to have renal benefits in a rat model of aggressive CKD and hypertension (5/6 renal ablation). In that study, it was shown to reduce systemic arterial pressure, proteinuria, and glomerulosclerosis, as well as oxidative stress and renal tissue inflammation. Those findings prompted us to develop the present study, the main objective of which was to evaluate the impact of Brazilian green propolis extract on proteinuria reduction and renal function in individuals with CKD.

## Methods

### Study design

This was a randomized, double-blind, placebo-controlled clinical trial. The study was conducted according to the principles of the Declaration of Helsinki and was approved by the Ethics Committee for Analysis of Research Projects of the *Hospital das Clínicas da Faculdade de Medicina da Universidade de São Paulo* (HC-FMUSP, University of São Paulo School of Medicine *Hospital das Clínicas*; Registration no. 54326916.4.0000.0068). The Trial was registered in ClinicalTrials.gov (identifier NCT02766036). All participating patients gave written informed consent.

### Design overview

**Fig. 1:**
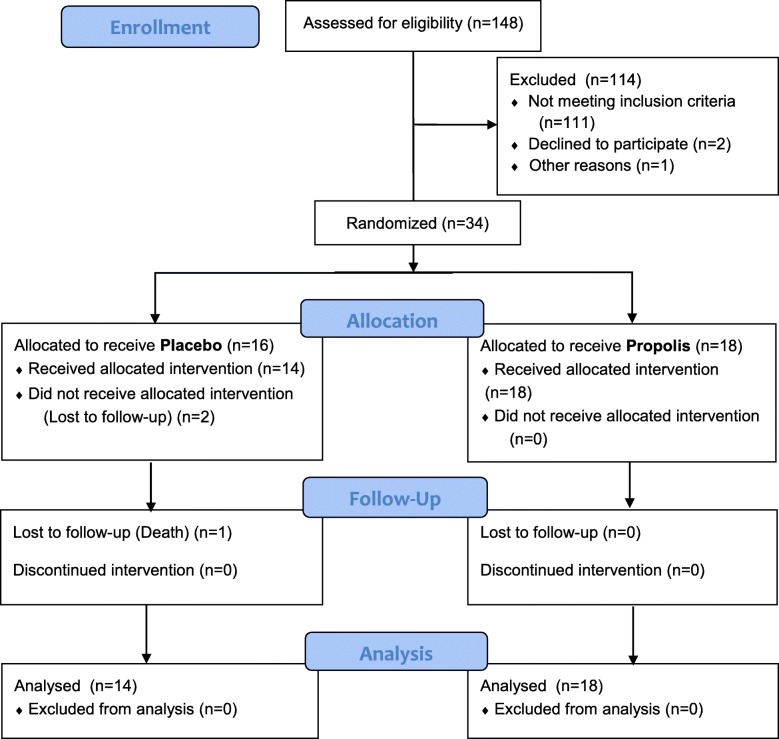
Consolidated Standards of Reporting Trials diagram showing the recruitment and follow-up of patients

Of 148 patients evaluated, 37 were deemed eligible to be followed for 3 months (a run-in phase) for the collection of data, evaluation of the stability of the estimated GFR (eGFR), and monitoring of proteinuria. The eGFR was determined with the Modification of Diet in Renal Disease formula. The eGFR is expressed in milliliters per minute per 1.73 m^2^. Of the 37 eligible patients, 32 were randomized to receive Brazilian green propolis extract (*n* = 18) or a placebo (*n* = 14) for 12 months. We used stratified randomization based on the factors age, ACE inhibitor or ARB use, the presence of type 2 diabetes, proteinuria and creatinine levels. The study flow diagram is shown in Fig. 1. Randomization was performed by an external investigator who was not involved in the care or follow-up of the patients. Patients were selected from among those under treatment at the HC-FMUSP Nephrology Outpatient Clinic. The patients were followed for 12 months, after which the blinding was broken.

### Participants

The study included patients between 18 and 90 years of age who had been diagnosed with CKD caused by diabetes or of another etiology, with an eGFR of 25–70 ml/min per 1.73m^2^ and proteinuria (defined as urinary protein excretion > 300 mg/day), together with micro- or macro-albuminuria, defined as a urinary albumin-to-creatinine ratio (UACR) > 30 mg/g urinary creatinine (uCr) and > 300 mg/g uCr, respectively. Kidney transplant recipients were excluded, as were pregnant women, patients with neoplasia, and patients with glomerulopathy who were receiving immunosuppressive therapy. The baseline characteristics of both groups are shown in Table 1.

**Table 1:** Baseline characteristics of patients with chronic kidney disease, treated with Brazilian green propolis or receiving a placebo

Characteristic	Placebo, *n* = 14	Propolis, *n* = 18	*P*
Age, yr, mean ± SD	61.50 ± 10.77	61.39 ± 10.47	0.97
Men, *n* (%)	7 (50.0)	11 (61.1)	0.72
Ethnicity, *n* (%)			0.84
White	6 (42.9)	6 (33.3)	
Black	5 (35.7)	7 (38.9)	
Mixed	3 (21.4)	5 (27.8)	
Cause of CKD, *n* (%)			
Diabetes	5 (35.7)	6 (33.3)	0.99
Hypertension	5 (35.7)	10 (66.6)	0.30
Glomerulopathy	2 (14.3)	0 (0)	0.18
Other	2 (14.3)	2 (11.1)	0.99
BMI (kg/m^2^), mean ± SD	27.29 ± 6.72	30.58 ± 6.42	0.17
Blood pressure (mmHg), mean ± SD			
Systolic	138.4 ± 18.11	142.2 ± 25.42	0.61
Diastolic	80.29 ± 10.5	85.33 ± 17.49	0.32
Creatinine (mg/dl), mean ± SD	1.89 ± 0.54	1.81 ± 0.47	0.69
eGFR^a^ (ml/min per 1.73 m2), mean ± SD	34.93 ± 1488	36.89 ± 11.5	0.68
Proteinuria (mg/day), mean (95% CI)	1097 (806 to 1493)	960 (677 to 1361)	0.57
UACR (mg/g uCr), mean ± SD			
All patients	1016.0 ± 740.6	870.3 ± 1010	0.50
Patients with diabetes^b^	1261.0 ± 1213.0	981.0 ± 709.8	0.66
HbA1c (%), mean ± SD			
All patients	6.57 ± 1.72	6.24 ± 1.21	0.54
Patients with diabetes^b^	8.14 ± 0.89	7.36 ± 1.31	0.27
25(OH)D (ng/ml), mean ± SD	26.29 ± 6.81	30.3 ± 10.82	0.20
Serum uric acid (mg/dl), mean ± SD	7.27 ± 0.66	6.67 ± 1.11	0.06
HDL (mg/dl), mean ± SD	51.79 ± 13.97	51.17 ± 12.84	0.89
Urinary MCP-1 (pg/mg uCr), mean ± SD	78.47 ± 89.99	94.84 ± 79.01	0.62
Antihypertensive drugs, *n* (%)			
ACE inhibitor or ARB	11 (78.6)	12 (66.7)	0.69
Beta-blocker	8 (57.1)	12 (66.7)	0.71
Calcium-channel blocker	6 (42.9)	9 (50.0)	0.73
Diuretic	8 (57.1)	10 (55.6)	0.99
Others	6 (42.9)	7 (38.9)	0.99
Statin, *n* (%)	11 (78.6)	15 (83.3)	0.99
Allopurinol, *n* (%)	9 (64.3)	11 (61.1)	0.99

*BMI*, body-mass index, *eGFR* estimated glomerular filtration rate, *uCr* urinary creatinine, *UACR* urinary albumin-to-creatinine ratio, *CKD* chronic kidney disease, *HbA1c* glycated hemoglobin, *HDL* high-density lipoprotein, *25(OH)D* 25-hydroxyvitamin D, *MCP-1* monocyte chemoattractant protein-1, *ACE* angiotensin-converting enzyme, *ARB* angiotensin receptor blocker

^a^Estimated according to the four-variable Modification of Diet in Renal Disease formula

^b^*n* = 6 in propolis group and *n* = 5 in placebo group

### Primary and secondary endpoints

The primary endpoint was a reduction in proteinuria. The secondary endpoint was a change in the eGFR over the follow-up period. Other measures included albuminuria, blood pressure, and the urinary level of monocyte chemoattractant protein-1 (MCP-1), which is a marker of inflammation. To assess safety, we measured markers of hepatic, muscle, and pancreatic injury, including alanine aminotransferase, aspartate aminotransferase, total bilirubin, creatine kinase, and amylase. Throughout the study, we also monitored patients to identify any adverse events or reactions.

### Characterization of the Propolis extract

Although there is no guarantee that natural products will be identical from lot to lot, a standardized green propolis extract has been proposed and has proven reproducible, on the basis of a set of chemical markers and antimicrobial activity [19]. That extract (EPP-AF; Apis Flora Indl. Coml. Ltda, Ribeirão Preto, Brazil), which is composed mainly of the green propolis found in southeast Brazil, was selected for use in the present study. To characterize the extract, we used high-performance liquid chromatography, with a diode array detector, as previously described by Berretta et al. [19] and depicted in Additional file 1. To ensure uniformity, all of the propolis tablets administered were from the same lot (no. 190000116, produced in Dec 2016). The daily dose of propolis provided 35.5 mg of total flavonoids (expressed as quercetin equivalents) and 77.96 mg of total phenolic compounds (expressed as gallic acid equivalents).

### Treatment

Patients in the propolis group received EPP-AF propolis at a dose of 500 mg/day (4 tablets of 125 mg each, divided into 2 daily doses). The chosen dose of propolis was based on studies that had used similar doses without observing adverse effects [24, 25]. Patients in the placebo group received an identical number of pills containing 500 mg/day of placebo (4 tablets of 125 mg each, divided into 2 daily doses). The labeling was identical for both groups. In both cases, the packaging that housed the tablets was opaque and had a security system to prevent undue opening. All of the tablets were coated and had the same organoleptic characteristics, so that the researchers involved in the care of the patients could not distinguish between the propolis and the placebo. The patients received standard treatment for the control of their comorbidities. The baseline dosages of ACE inhibitors or ARBs were maintained throughout the study. For other blood pressure disorders, other classes of antihypertensive drugs were used.

### Measurements

Patients were evaluated at baseline, every 2 months for the first 6 months, and every 3 months for the next 6 months. Adherence was assessed indirectly, through interviews, and directly, through tablet counts. For hypertensive patients, home blood pressure monitoring was performed.

At each medical appointment, anthropometric parameters (weight, height, and waist circumference) were measured. Prior to each medical appointment, we obtained three blood pressure measurements, with a two-minute interval between each measurement, using a mercury sphygmomanometer and a cuff of adequate size, in a calm, quiet environment, without the physician present. The mean of the three measurements was used for analysis.

### Laboratory assessments

All biochemical tests were analyzed at the HC-FMUSP Central Laboratory, a certified laboratory that follows international standards. The urinary albumin concentration was determined by immunoturbidimetry. The 24-h urine samples were collected by the patients, who were instructed in the proper procedure by the medical and laboratory team. Each sample was collected in an appropriate, standard, sterile plastic bottle without preservative.

At baseline and month 12, simple urine samples were also collected for MCP-1 analysis. Those samples were immediately put on ice, centrifuged at 0 °C, and stored at − 80 °C until use. Urinary MCP-1 was measured by enzyme-linked immunosorbent assay (Human CCL2/MCP-1 Quantikine ELISA Kit; R & D Systems, Minneapolis, MN, USA), was normalized to uCr (measured in the same urine sample), and is expressed in pg/mg uCr. Other parameters were measured with conventional laboratory techniques.

### Sample size calculation

To calculate the sample size, we estimated an effect of differences in relation to a mean 12-month level of proteinuria of 500 mg/day using t-test, and assumed a standard deviation of approximately 460 mg/day [26]. Thus, we determined that a sample of 18 patients per group (*N* = 36), at a 5% level of significance, would have a power of 90%.

### Statistical analyses

Continuous variables are expressed as mean and standard deviation (SD) or as mean and 95% confidence interval (95% CI). Categorical variables are expressed as absolute and relative frequencies. To compare the baseline characteristics between the two groups, Student’s t-test or χ2 test were used for parametric and non-parametric variables respectively. We used intention-to-treat analyses for the primary and secondary endpoints. Each variable was evaluated by means of mixed linear regression models considering intercept random effects for the individual and fixed effects of time, group, and the interaction between the two. For the variables proteinuria, alanine aminotransferase, aspartate aminotransferase, total bilirubin, creatine kinase, and MCP-1, the assumption of normal distribution was not satisfied, and they were therefore adjusted in generalized linear mixed-effect models considering the gamma distribution for the dependent variable. In the subgroups of diabetic patients, we evaluated albuminuria with Wilcoxon signed rank test. The analysis was performed with the program R, version 3.4.1 (R Core Team, 2017). For all tests, the level of significance was set at 5%.

## Results

### Study population

At baseline, the demographic, clinical, and biochemical characteristics were similar between the propolis and placebo groups (Table 1). We initially screened 148 patients and identified 37 who were eligible to participate in the 3-month run-in phase, during which three patients were excluded (one died of unknown causes and two declined to participate). At 9 months into the one-year study period, one patient in the placebo group died from abdominal sepsis. Data for that patient were included in intention-to-treat analyses of the primary and secondary outcomes. Two patients in the placebo group patients were lost to follow-up. The flow diagram is shown in Fig. 1.

### Primary efficacy analyses

**Fig. 2:**
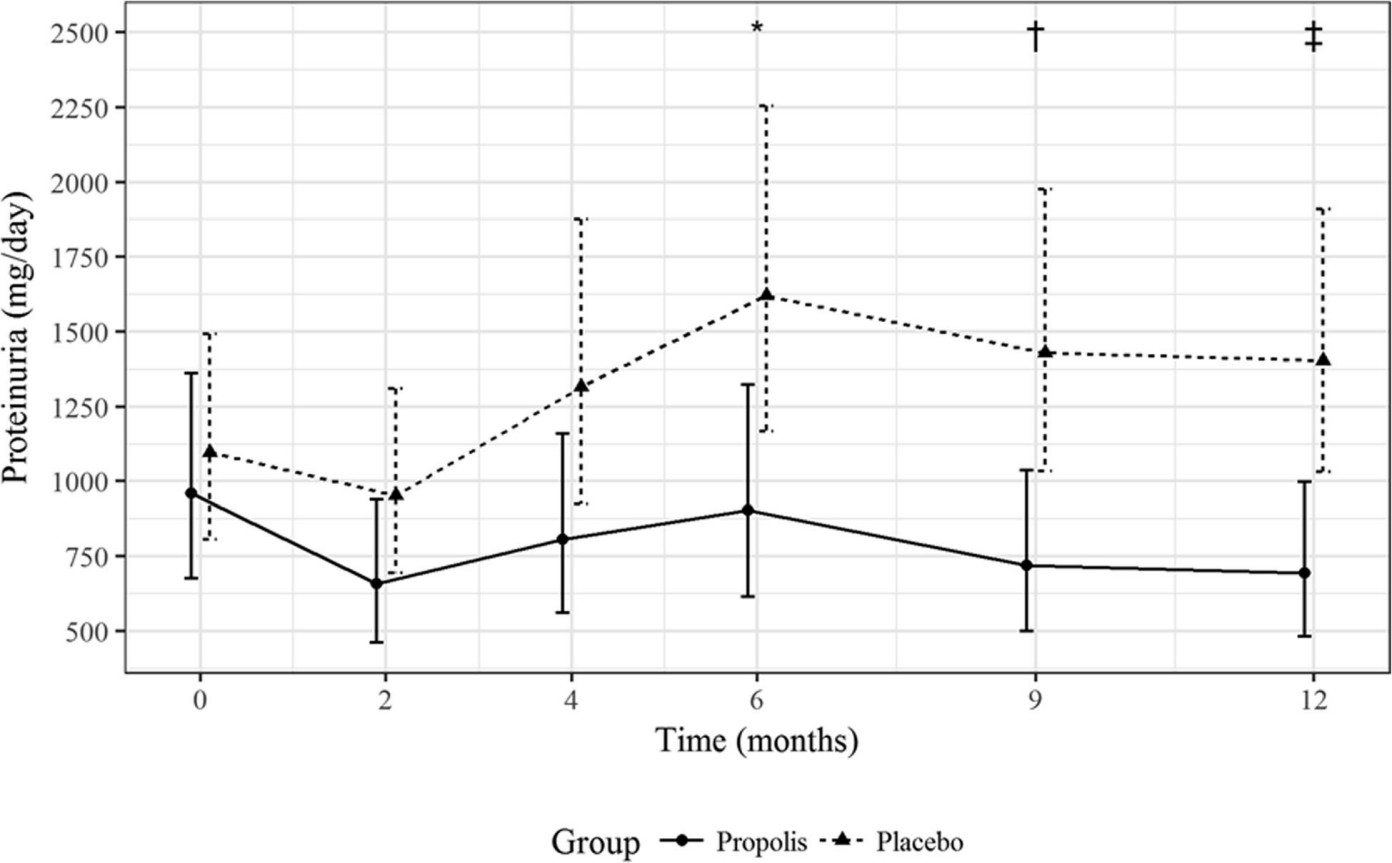
Changes in proteinuria (mg/day) during follow up. Values presented as mean and 95% CI for each time point according to the mixed-effect linear regression model. *P = 0.023 vs. placebo; †*P* = 0.006 vs. placebo; ‡*P* = 0.004 vs. placebo

At the end of the study, proteinuria was significantly lower in the propolis group than in the placebo group— 695 mg/24 h (95% CI, 483 to 999) vs. 1403 mg/24 h (95% CI, 1031 to 1909); *P* = 0.004—as can be seen in Fig. 2. There was no difference between the two groups at the beginning of the study, propolis group — 960 mg/24 h (95% CI, 677 to 1361) at baseline and placebo group — 1097 mg/24 h (95% CI, 807 to 1493) at baseline; *P* = 0.57. The difference between the two groups, in terms of the mean level of proteinuria, was evident by month 2 and became significant by month 6.

### Secondary outcomes

**Fig. 3:**
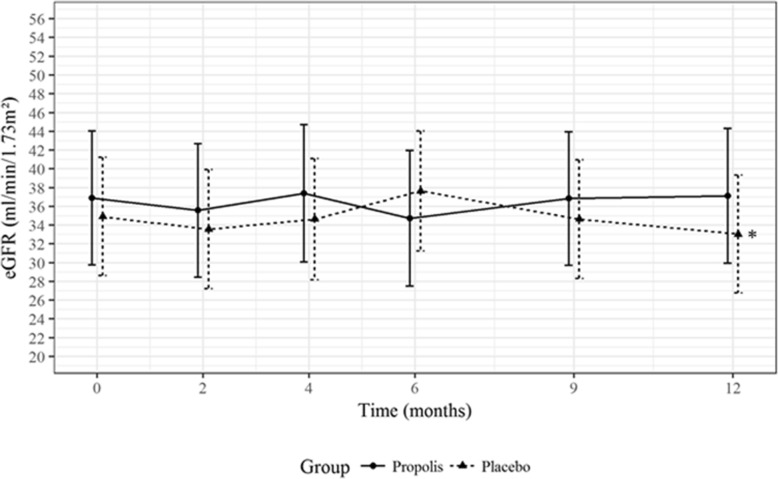
Changes in estimated glomerular filtration rate (eGFR, ml/min per 1.73 m2) during follow up. Values presented as mean and 95% CI for each time point. *P = 0.40 vs. placebo

At the end of follow-up (month 12), there was no statistical difference between the propolis and placebo groups in terms of the eGFR—37 ml/min per 1.73m^2^ (95% CI, 30 to 44) vs. 33 ml/min per 1.73m^2^ (95% CI, 27 to 39); *P* = 0.40—as shown in Fig. 3.

**Fig. 4:**
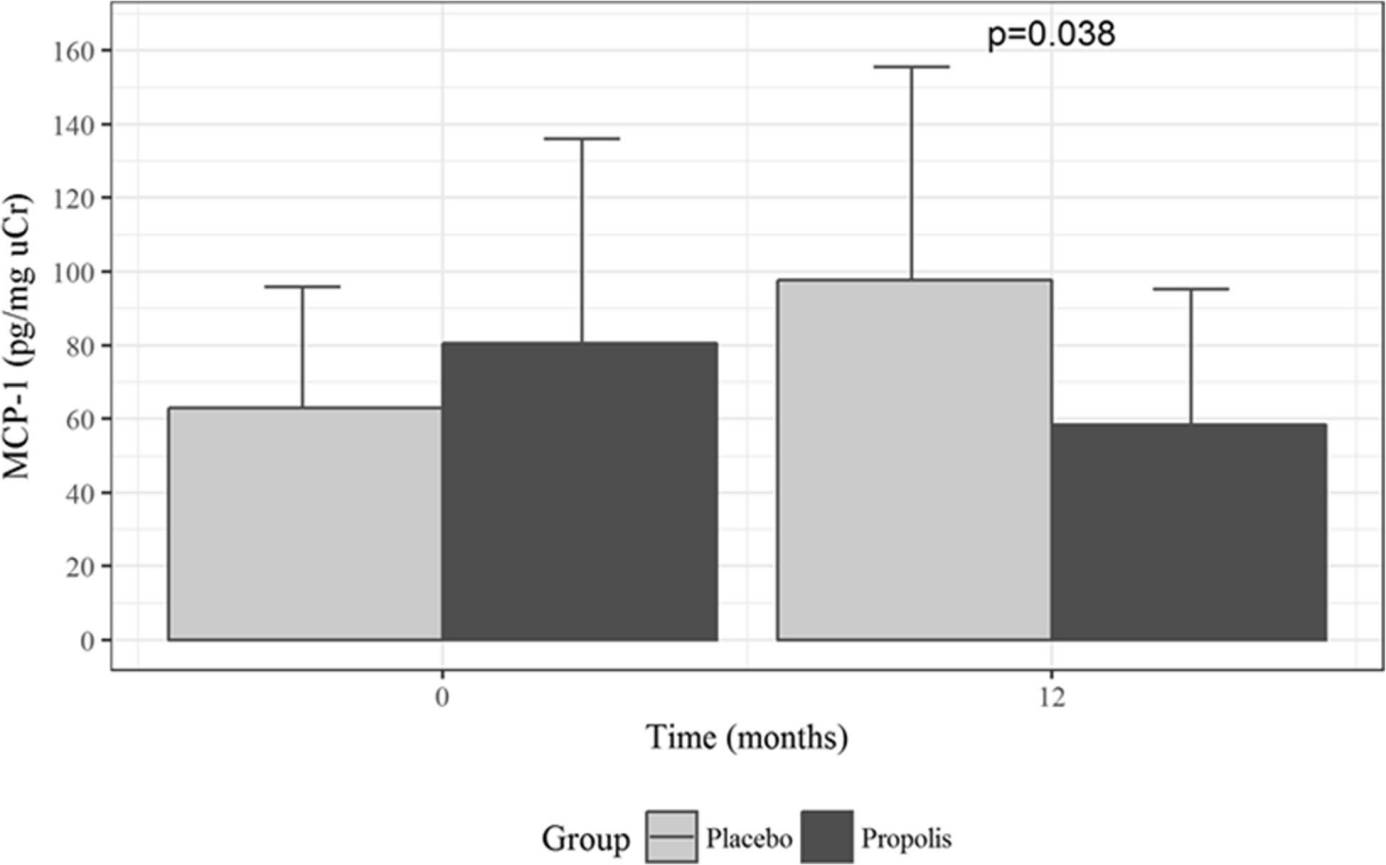
Changes in urinary monocyte chemoattractant protein-1 (MCP-1, pg/mg urinary creatinine) during follow up. Values presented as mean and 95% CI for each time point. *P = 0.038 vs. placebo

Figure 4 shows the mean urinary MCP-1 levels, which were significantly lower at month 12 in the propolis group than in the placebo group—58 pg/mg uCr (95% CI, 36 to 95) vs. 98 pg/mg uCr (95% CI, 62 to 155); *P* = 0.038.

**Fig. 5:**
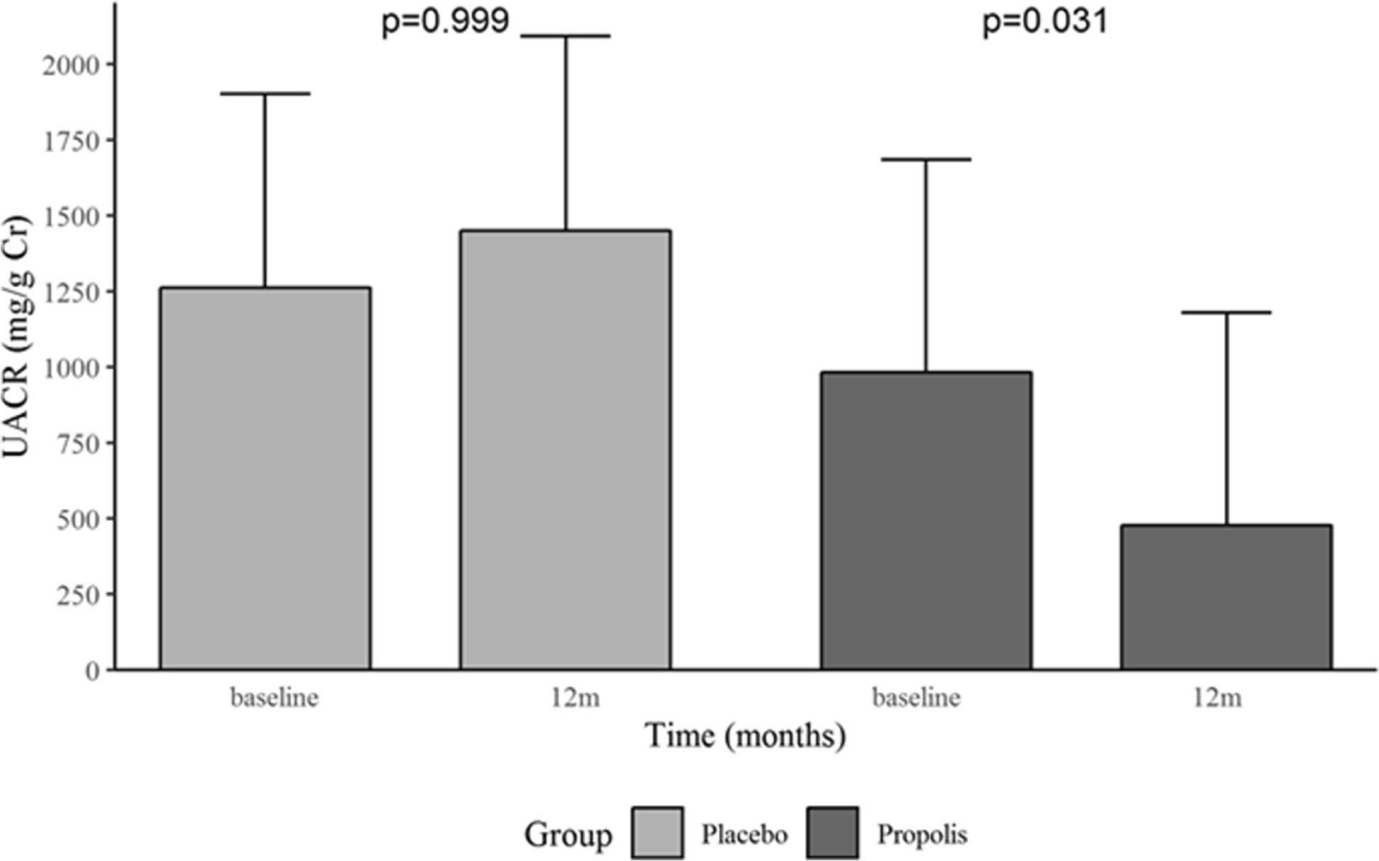
Urinary albumin-to-creatinine ratio (UACR) in the subgroups of patients with type 2 diabetes, at baseline and 12 months (12 m). Propolis (*n* = 6) and Placebo (*n* = 5). Values presented as mean and 95% CI

Within the subgroup of patients with CKD caused by diabetes, those who received propolis showed a significant reduction in the mean UACR (Fig. 5), from 981 mg/g uCr (95% CI, 223 to 1739) at baseline to 476 mg/g uCr (95% CI, − 282 to 1235) at month 12 (*P* = 0.031), whereas the mean UACR increased among those who received the placebo, from 1261 mg/g uCr (95% CI, 569 to 1953) at baseline to 1451 mg/g uCr (95% CI, 758 to 2143) at month 12 (*P* = 0.999). Nevertheless, at month 12, the difference between those who received propolis and those who received the placebo was not significant (*P* = 0.259).

**Fig. 6:**
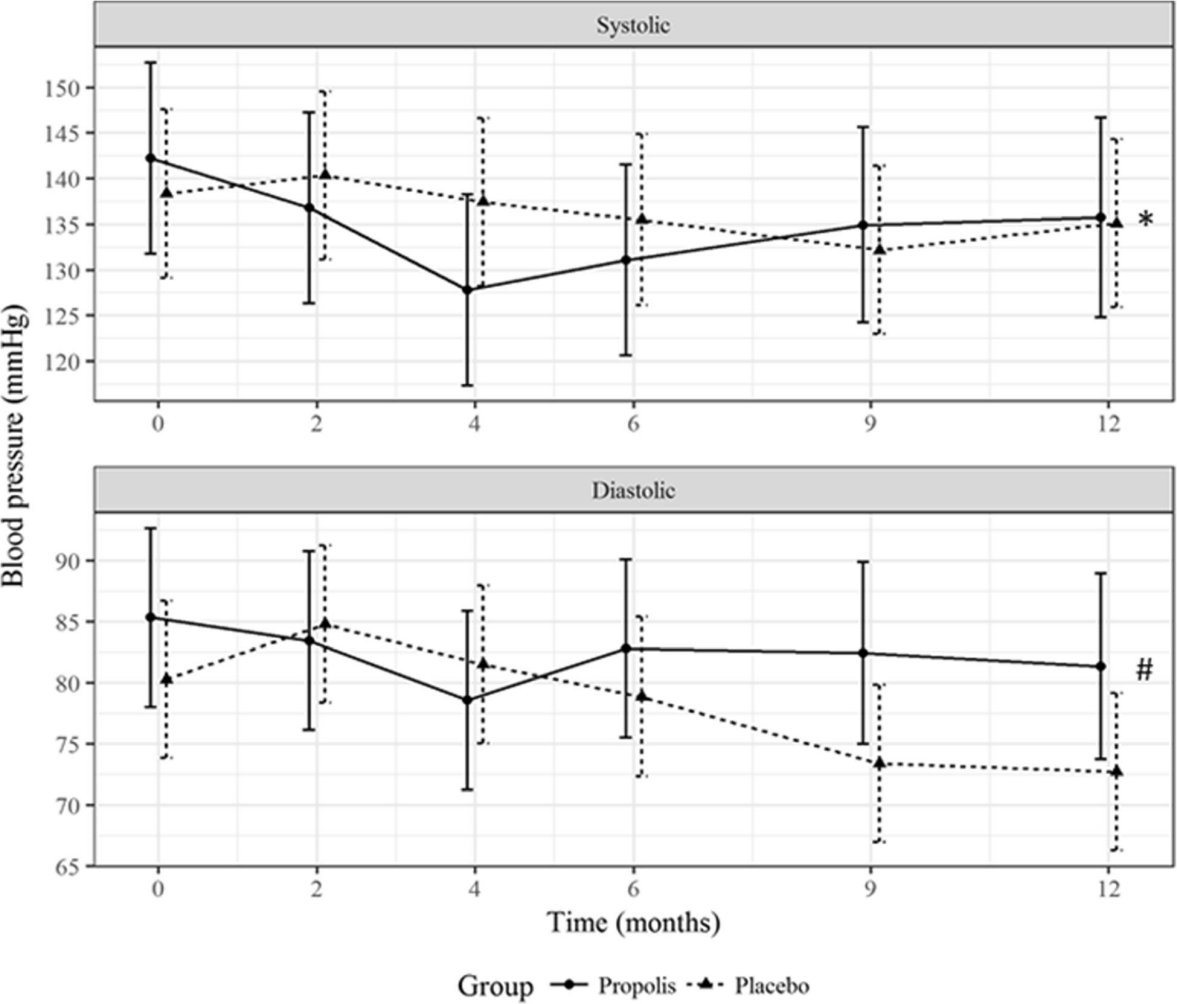
Changes in systolic and diastolic blood pressure, in mmHg. Values presented as mean and 95% CI for each time point. *P = 0.93 vs. placebo; #*P* = 0.089 vs. placebo

The mean systolic and diastolic blood pressures remained stable throughout the follow-up period, without statistical differences between the groups (Fig. 6). At month 12, the mean systolic blood pressure in the propolis and placebo groups was 135 mmHg (95% CI, 125 to 145) and 135 mmHg (95% CI, 126 to 144), respectively (*P* = 0.93), compared with 81 mmHg (95% CI, 74 to 89) and 73 mmHg (95% CI, 66 to 79), respectively, for the mean diastolic blood pressure (*P* = 0.089).

Glycated hemoglobin (HbA1c) did not differ between the groups during follow-up. At 12 months, the mean HbA1c in the propolis and placebo groups was 6.35% (95% CI, 5.59 to 7.12) and 7.32% (95% CI, 5.80 to 8.87), respectively (*P* = 0.20). Among the patients with type 2 diabetes, at the end of the study, the means those in the propolis and placebo groups showed a mean HbA1c of 7.38% (95% CI, 5.50 to 9.25) and 8.13% (95% CI, 6.87 to 9.41), respectively (*P* = 0.14).

The markers of hepatic and muscle damage did not change significantly during the 12 months of treatment (Table 2). The difference between the propolis group and the placebo group, in terms of the mean level of amylase, a marker of pancreatic injury—94.3 U/L (95% CI, − 45.3 to 234.5) vs. 105.7 U/L (95% CI, 100.5 to 110.8)—was not significant (*P* = 0.76). Given the reference range for amylase (28–100 U/L), that finding demonstrates the safety of propolis at the dose administered. None of the participants reported any adverse effects or allergic reactions during the treatment.

**Table 2:** Biochemical safety data^a^

Variable	Placebo, *n* = 14			Propolis, *n* = 18			p^b^
	Time point			Time point			
	Baseline	6 months	12 months	Baseline	6 months	12 months	
AST (U/L)^c^	25.2 ± 11.6	27 ± 16.3	36 ± 28	18.3 ± 4.3	19.3 ± 5.8	18.9 ± 6.7	0.34
ALT (U/L)^d^	25 ± 9.2	25.2 ± 14.7	41.4 ± 28.3	17.3 ± 6.2	16.4 ± 5.3	17.5 ± 7.3	0.02
TB (mg/dl)^e^	0.7 ± 0.4	0.7 ± 0.4	0.6 ± 0.3	0.5 ± 0.3	0.4 ± 0.2	0.4 ± 0.2	0.62
CK (U/L)^f^	126.8 ± 64.1	124.2 ± 64.3	108.1 ± 54.6	145.2 ± 83.5	140.9 ± 95.5	145.5 ± 100.9	0.16

AST, aspartate aminotransferase; ALT, alanine aminotransferase; CK, (plasma) creatine kinase; TB, total bilirubin.

^a^Data expressed as mean ± SD

^b^Baseline vs. 12 months

^c^Reference values: ≤41 U/L for men and ≤ 31 U/L for women

^d^Reference values: ≤64 U/L for men and ≤ 23 U/L for women

^e^Reference range: 0.2–1.0 mg/dl

^f^Reference values: ≤190 U/L for men and ≤ 167 U/L for women

## Discussion

In the present study, we selected patients with CKD of diverse etiologies, most with a moderate loss of renal function. We observed significantly (≈30%) lower proteinuria in patients treated for 12 months with green propolis than in those receiving a placebo, that difference becoming significant by month 6 and persisting until the end of treatment, regardless of the etiology of the CKD.

Mechanisms related to the possible antiproteinuric effect of propolis have yet to be fully elucidated. In an experimental study involving hypertensive rats with CKD and proteinuria (5/6 renal ablation model), the authors observed a reduction in proteinuria, which was related to lower urinary oxidative stress and reduced renal infiltration by macrophages [23]. It has recently been shown that chrysin, one of the flavonoids present in propolis, reduces the podocyte apoptosis induced by exposure to high glucose concentrations, as well as having an antiproteinuric effect and reducing glomerular injury, in rats with diabetes [27]. In the present study, we did not observe significant differences in blood pressure or eGFR between the propolis and placebo groups. Therefore, we believe that the antiproteinuric effect of propolis was not due to changes in systemic hemodynamic parameters.

Experimental studies have demonstrated that the use of propolis can reduce blood pressure, the proposed mechanisms of action including a nitric oxide pathway, acetylcholine-induced vasodilation, and the antioxidant activity of the propolis itself [23, 28–30]. Despite such experimental evidence, we observed no propolis-related difference in blood pressure over the course of the study. That might be explained by the fact that the hypertensive patients evaluated in our study were under treatment with antihypertensive medications.

Within our subgroup of patients with CKD caused by diabetes, those who received propolis showed a significant reduction in albuminuria (i.e., the mean UACR) over the course of the study. There is some evidence that propolis has a hypoglycemic effect [27], and it is therefore noteworthy that the apparently propolis-induced reduction in proteinuria occurred independently of significant variations in the glycemic index during treatment.

We observed no significant difference in plasma creatinine between the two groups evaluated in the present study. However, the 12-month observation period might have been too short to evaluate the progression of CKD through the measurement of creatinine, which has well-known limitations. However, because creatinine levels remained stable throughout the treatment period, during which there was a reduction in proteinuria, we can suggest that the antiproteinuric effect of propolis is independent of variations in glomerular filtration. In addition, because proteinuria is a recognized marker of glomerular injury, as well as being associated with renal disease progression and higher cardiovascular risk, its reduction is considered an extremely positive factor in the assessment of the effectiveness of a potentially renoprotective drug.

The cytokine MCP-1 promotes the recruitment of monocytes and their transformation into macrophages. Its elimination through urine signals inflammatory aggression in renal tissue, and a recent study showed that MCP-1 levels correlate positively with CKD progression [31]. In addition, experimental studies have shown that the use of a MCP-1 receptor blocker suppresses inflammation and reduces glomerulosclerosis, as well as that the stimulus for the nuclear synthesis of MCP-1 is associated with oxidative stress pathways, nuclear factor-kappa B transcription factor, and protein kinase C [31–34]. Few clinical trials have used urinary MCP-1 in the evaluation of proteinuria [35–37]. In our study, the group receiving propolis showed a progressive reduction in urinary MCP-1 over the 12 months of treatment, which could represent one of the mechanisms of propolis in the reduction of proteinuria.

Although propolis has been used in folk medicine for hundreds of years, there have been sporadic reports of allergic phenomena, including a condition similar to acute interstitial nephritis [38]. In the present study, there were no patient complaints related to the use of propolis, nor did we observe any biochemical abnormalities that would indicate toxicity. It should also be borne in mind that allergic phenomena are observed even with medications traditionally used in the treatment of nephropathies, such as ACE inhibitors. The possibility of that propolis components will interact with cytochrome P450 isoenzymes is considered low [39]. Two patients in our propolis group were using the anticoagulant warfarin and did not require any adjustment in the dose or show significant changes in the international normalized ratio.

Our study has some limitations. It was a single-center involving and a relatively small sample, that needs further investigation in other and larger populations. A relatively short follow-up period was adequate to evaluate changes in proteinuria, but too short to analyze changes in glomerular filtration rate. However, those limitations were at least partially offset by the randomized, double-blind study design, the inclusion of patients with CKD of different etiologies, and the evaluation of other variables related do CKD progression.

## Conclusions

In conclusion, treatment with Brazilian green propolis appears to be capable of reducing proteinuria significantly in patients with CKD of any etiology and moderate renal dysfunction. The antiproteinuric effect of propolis seems to be independent of variations in blood pressure and GFR. We also observed a significant reduction in urinary excretion of MCP-1 after treatment with propolis. These data indicate the therapeutic potential of Brazilian green propolis, opening perspectives for its use as a natural coadjuvant in the treatment of the proteinuric forms of renal diseases.

## Additional file

Additional file1: Chemical characterization of the standardized propolis extract (EPP-AF) used in this study by high-performance liquid chromatography (HPLC). The propolis extracts were analyzed by HPLC using a Shimadzu apparatus equipped with a CBM-20A controller, a LC-20AT quaternary pump, a SPD-M 20A diode-array detector, and Shimadzu LC solution software, version 1.21 SP1. A Shimadzu Shim-Pack CLC-ODS column (4.6 × 250 mm, particle diameter of 5 μm, pore diameter of 100 Å) was used. The mobile phase consisted of methanol (B) and a water-formic acid solution (0.1% *v*/v), pH 2.7 (A). The method consisted of a linear gradient of 20–95% of B over a period of 77 min at a flow rate of 0.8 ml/min. Detection was set at 275 nm. Propolis extracts were diluted with 5 ml of methanol (HPLC grade) in 10-ml volumetric flasks, subjected to sonication for 10 min, and filled to volume with Milli-Q water. The samples were filtered through a 0.45-μm filter before analysis. The commercially produced extract was kindly provided by the Apis Flora Company, Ribeirão Preto, Brazil (Patent no. PI 0405483–0, published in the *Revista de Propriedade Industrial* n. 1778 from 01/02/2005). (TIF 99 kb)
