# Supplementary figures and images for: Effects of Brazilian green propolis on proteinuria and renal function in patients with chronic kidney disease: a randomized, double-blind, placebo-controlled trial
Source: BMC Nephrol. 2019 Apr 25;20:140. doi: 10.1186/s12882-019-1337-7 (PMC6485062; doi:10.1186/s12882-019-1337-7)

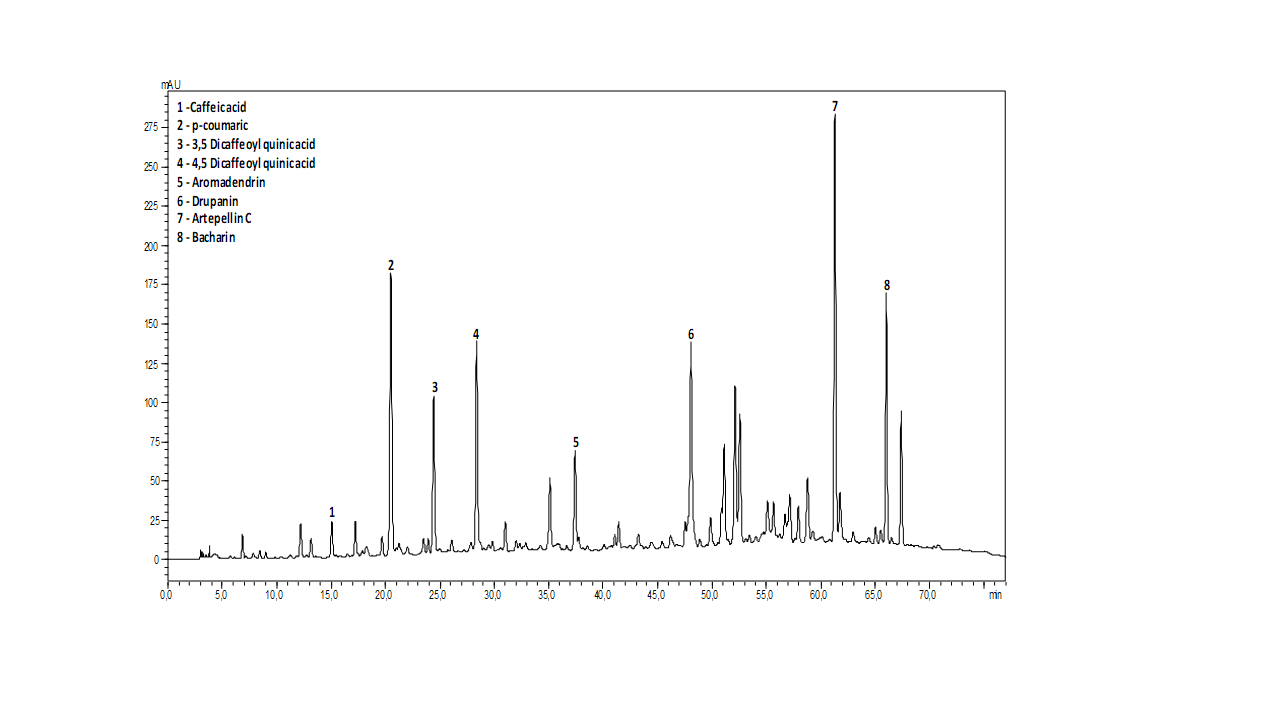

Supplement: Supplementary file 1 — Chemical characterization of the standardized propolis extract (EPP-AF) used in this study by high-performance liquid chromatography (HPLC). The propolis extracts were analyzed by HPLC using a Shimadzu apparatus equipped with a CBM-20A controller, a LC-20AT quaternary pump, a SPD-M 20A diode-array detector, and Shimadzu LC solution software, version 1.21 SP1. A Shimadzu Shim-Pack CLC-ODS column (4.6 × 250 mm, particle diameter of 5 μm, pore diameter of 100 Å) was used. The mobile phase consisted of methanol (B) and a water-formic acid solution (0.1% v/v), pH 2.7 (A). The method consisted of a linear gradient of 20–95% of B over a period of 77 min at a flow rate of 0.8 ml/min. Detection was set at 275 nm. Propolis extracts were diluted with 5 ml of methanol (HPLC grade) in 10-ml volumetric flasks, subjected to sonication for 10 min, and filled to volume with Milli-Q water. The samples were filtered through a 0.45-μm filter before analysis. The commercially produced extract was kindly provided by the Apis Flora Company, Ribeirão Preto, Brazil (Patent no. PI 0405483–0, published in the Revista de Propriedade Industrial n. 1778 from 01/02/2005). (TIF 99 kb) [file 12882_2019_1337_MOESM1_ESM.tif]
